# COVID-19-Associated Encephalopathy: A Case Series Demonstrating the Rapid Deterioration of Mental Status and a Review of the Literature

**DOI:** 10.7759/cureus.76005

**Published:** 2024-12-19

**Authors:** Jacky Reny, Usman Siddiqui, Andrew Cox, Hussam Al Hennawi, Jamie Swanson, Arthur Siegel, Todd Goldberg

**Affiliations:** 1 Medicine, Sidney Kimmel Medical College, Philadelphia, USA; 2 Internal Medicine, Jefferson Abington Hospital, Abington, USA; 3 Geriatrics, Jefferson Abington Hospital, Abington, USA; 4 Palliative Care, Jefferson Abington Hospital, Abington, USA

**Keywords:** care disposition, cognitive impairment, covid-19, delirium, encephalopathy

## Abstract

Coronavirus disease 2019 (COVID-19), caused by severe acute respiratory syndrome coronavirus 2 (SARS-CoV-2), is known for its severe inflammatory response, often leading to multi-organ dysfunction. Among the less-recognized complications is COVID-19-associated encephalopathy, particularly in the elderly, where it contributes significantly to morbidity and mortality. This report explores the rapid neurocognitive decline observed in six hospitalized patients with COVID-19, with or without pre-existing neurological conditions. Each case highlights the challenges of managing complex clinical courses and emphasizes the importance of early, multidisciplinary intervention, including palliative care, to address the goals of care. Given the underreporting of COVID-19-associated encephalopathy, this case series underscores the need for increased awareness and specialized care to improve patient outcomes, particularly in older populations.

## Introduction

Coronavirus disease 2019 (COVID-19), caused by severe acute respiratory syndrome coronavirus 2 (SARS-CoV-2), has been increasingly recognized for its neurological complications, including encephalopathy and delirium [1]. The causes of COVID-19-associated encephalopathy are multifactorial, involving toxic, metabolic, ischemic, septic, and inflammatory pathways. Despite its high incidence, this condition remains underreported and poorly understood.

Studies indicate that neurological symptoms, such as altered mental status, occur in 7%-9% of hospitalized COVID-19 patients, with the incidence rising to 67% in Intensive Care Unit (ICU) cases [2,3]. Long-term cognitive issues, including confusion and inattention, can persist for months after recovery [4]. Dysphagia is another frequent complication, affecting up to 20.6% of patients and increasing the risk of aspiration pneumonia [5].

While the mechanisms remain unclear, neuroimaging and cerebrospinal fluid (CSF) analyses suggest both direct viral invasion and indirect inflammatory responses [6-8]. Despite emerging research, the clinical course and prognosis of COVID-19-associated encephalopathy remain uncertain [9]. This report aims to examine the clinical trajectories and outcomes of patients with COVID-19-associated encephalopathy, highlighting the importance of multidisciplinary care to improve patient outcomes.

## Materials and methods

In this case series, we will investigate the hospital course of patients admitted to the general medical floor, diagnosed with COVID-19 infection, and who were consulted by the palliative care team for concerns of altered mental status. The Institutional Review Board (IRB) evaluated human subjects' involvement in the proposed research, and, in accordance with Federal-Wide Assurance #00002109 to the U.S. Department of Health and Human Services, this research was expected per Exemption category: Category 4: Secondary Data and Tissue Use per 45 CFR 46.104(b). 

## Results

Case presentation

Case 1

**Figure 1:**
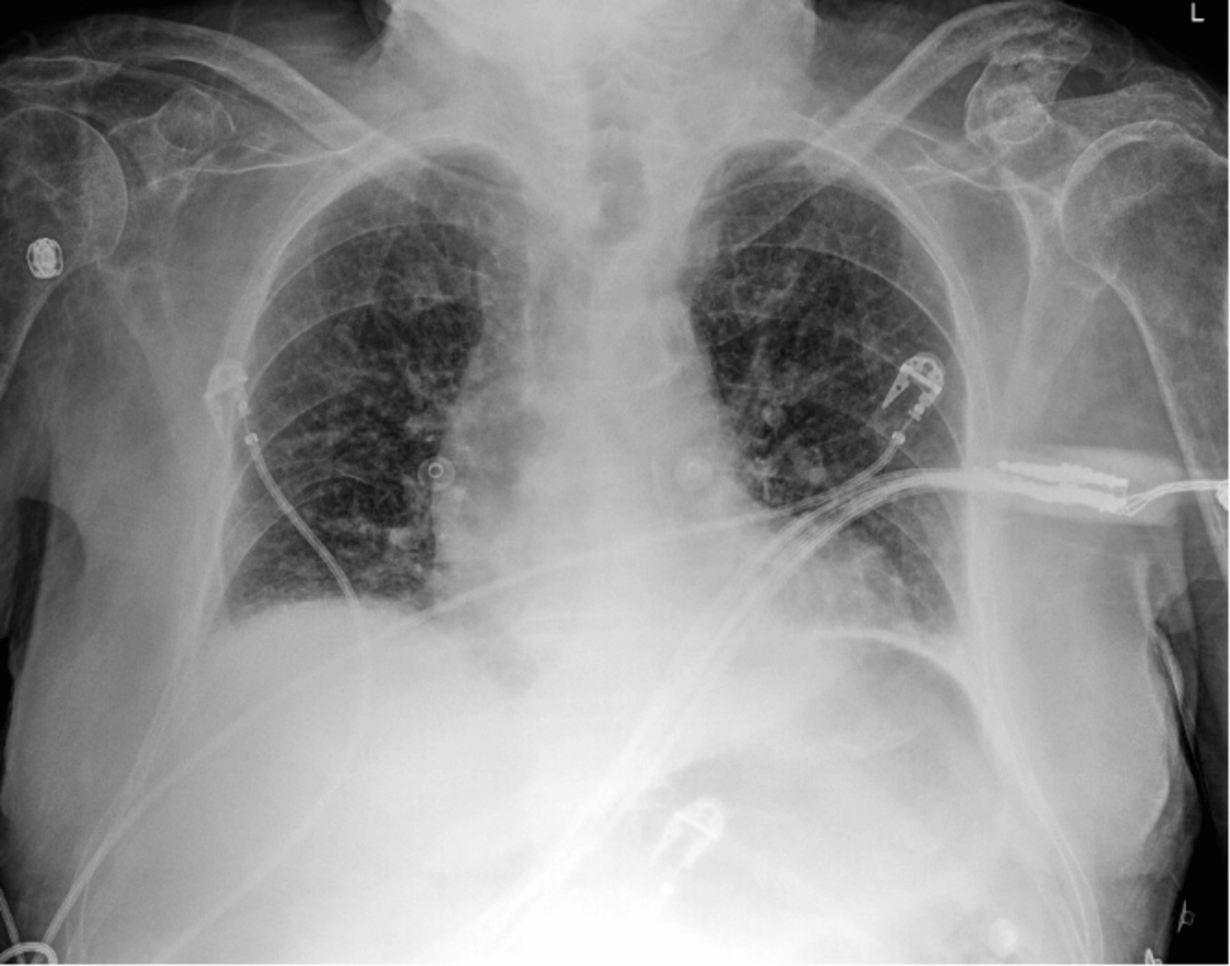
Chest X-ray showing mild edema, pulmonary venous congestion, and left basilar atelectasis.

A 94-year-old male patient with a medical history of prior stroke, resulting in residual right-sided hemiparesis and expressive aphasia, presented with worsening dyspnea and fever. Upon admission, SARS-CoV-2 RNA nucleic acid amplification test (NAAT) testing returned positive, and radiographic imaging showed evidence of mild pulmonary edema (Figure 1). Subsequently, the patient received a four-day course of remdesivir. On the day of admission, blood cultures were positive for *Enterococcus faecalis* and coagulase-negative *Staphylococcus*. The patient’s condition was managed with a combination of ceftriaxone and vancomycin, which was later switched to amoxicillin.

The patient’s clinical course was complicated by severe sepsis, characterized by tachycardia, tachypnea, fever, lactic acidosis, and transaminitis. He was placed on nothing-by-mouth (NPO) status on day 1, and a subsequent speech-language pathology (SLP) evaluation indicated acute moderate oropharyngeal dysphagia, in addition to chronic dysphagia, on day 2. He was initiated on a pureed diet. Further SLP evaluation on day 4 indicated no improvement in dysphagia, and the patient’s symptoms of dementia worsened over the duration of the hospitalization.

A palliative discussion was initiated with the patient’s family regarding goals of care, including wishes on feeding tube placement. After careful consideration, the decision was made to proceed with comfort-directed care, including a pureed diet to reduce the risk of aspiration and for energy conservation, as well as as-needed administration of Ativan and morphine. After six days of hospitalization, the patient was discharged to hospice on oral linezolid.

Case 2

A 91-year-old male patient with a medical history of Alzheimer’s dementia and diffuse large B-cell lymphoma presented with increased confusion from baseline and fatigue for one day. SARS-CoV-2 RNA NAAT testing returned positive upon admission, and radiographic imaging showed evidence of bilateral patchy opacities. The patient received a five-day course of remdesivir and a three-day course of dexamethasone, which was discontinued due to the development of psychosis. Empiric antibiotics were initiated for possible superimposed bacterial pneumonia, including cefepime, vancomycin, and levofloxacin.

**Figure 2:**
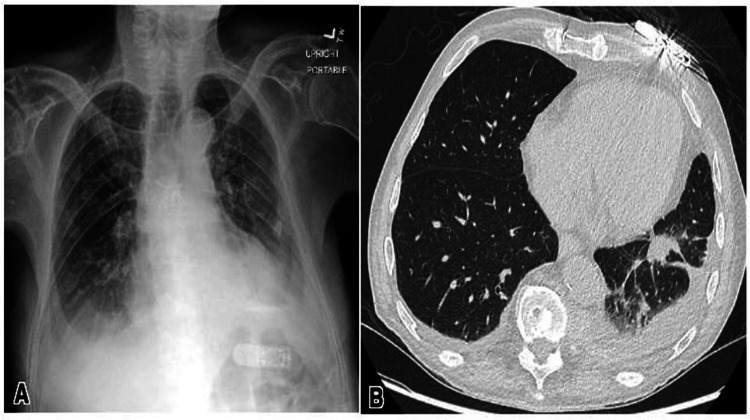
Chest X-ray showing mild edema, pulmonary venous congestion, and left basilar atelectasis (A). Computerized tomography of the chest showing left pleural effusion with left lower lobe opacities and calcified pleural plaques (B).

The patient’s computed tomography (CT) head was negative for acute intracranial abnormalities. Chest X-ray demonstrated mild edema, pulmonary venous congestion, and left basilar atelectasis (Figure 2A). The CT chest revealed bilateral pleural effusions with nonspecific left lower lobe opacities and calcified pleural plaques (Figure 2B). The patient’s clinical course was complicated by pancytopenia, newly diagnosed cardiomyopathy with an ejection fraction of 20%, and atrial fibrillation with a rapid ventricular response. He was on a thick liquid diet throughout the hospitalization. SLP evaluated him on hospital day 9 and concluded moderate oral dysphagia, moderate-to-severe pharyngeal dysphagia, and cricopharyngeal dysfunction. All of these findings were worsened from baseline.

In view of the patient’s terminal hematological malignancy and worsening dementia starting on hospital day 2, goals of care discussions were initiated with the family. His cognitive function remained decreased from baseline by the time of discharge. After careful consideration, the decision was made to initiate home hospice care with the continuation of as-needed morphine, lorazepam, haloperidol, and hyoscyamine. The patient was discharged home after 13 days of hospitalization, and, upon a follow-up call three months after discharge, the family informed a healthcare professional that the patient had died.

Case 3

An 87-year-old male patient with a history of late-onset Alzheimer’s disease presented with worsened weakness compared to his baseline after his wife found him on the floor next to his bed. Upon admission, SARS-CoV-2 RNA NAAT testing returned positive, while chest X-ray and urinalysis were unremarkable. The patient was subsequently started on intravenous (IV) remdesivir, and his home memantine was continued. During his hospitalization, the patient’s clinical course was complicated by a non-strangulated, non-obstructing inguinal hernia identified on imaging, which was deferred for outpatient surgical management.

On hospital day 1, the patient’s nurse noticed difficulty swallowing pills, prompting a subsequent SLP evaluation. Although the patient was on a regular, non-restricted diet at home, the SLP evaluation on hospital day 3 determined he had mild-to-moderate oropharyngeal dysphagia, leading to a transition to a thin liquid and minced/moist diet. The patient’s mental status continued to decline, with fluctuating orientation and minimal response to questions.

Given the worsening dementia noted on hospital day 1, goals of care discussions were initiated with the family. After careful consideration, the decision was made to continue care at a rehabilitation center, with plans for hospice if no improvement was observed. The patient was discharged to a rehab center after eight days of hospitalization.

Case 4

**Figure 3:**
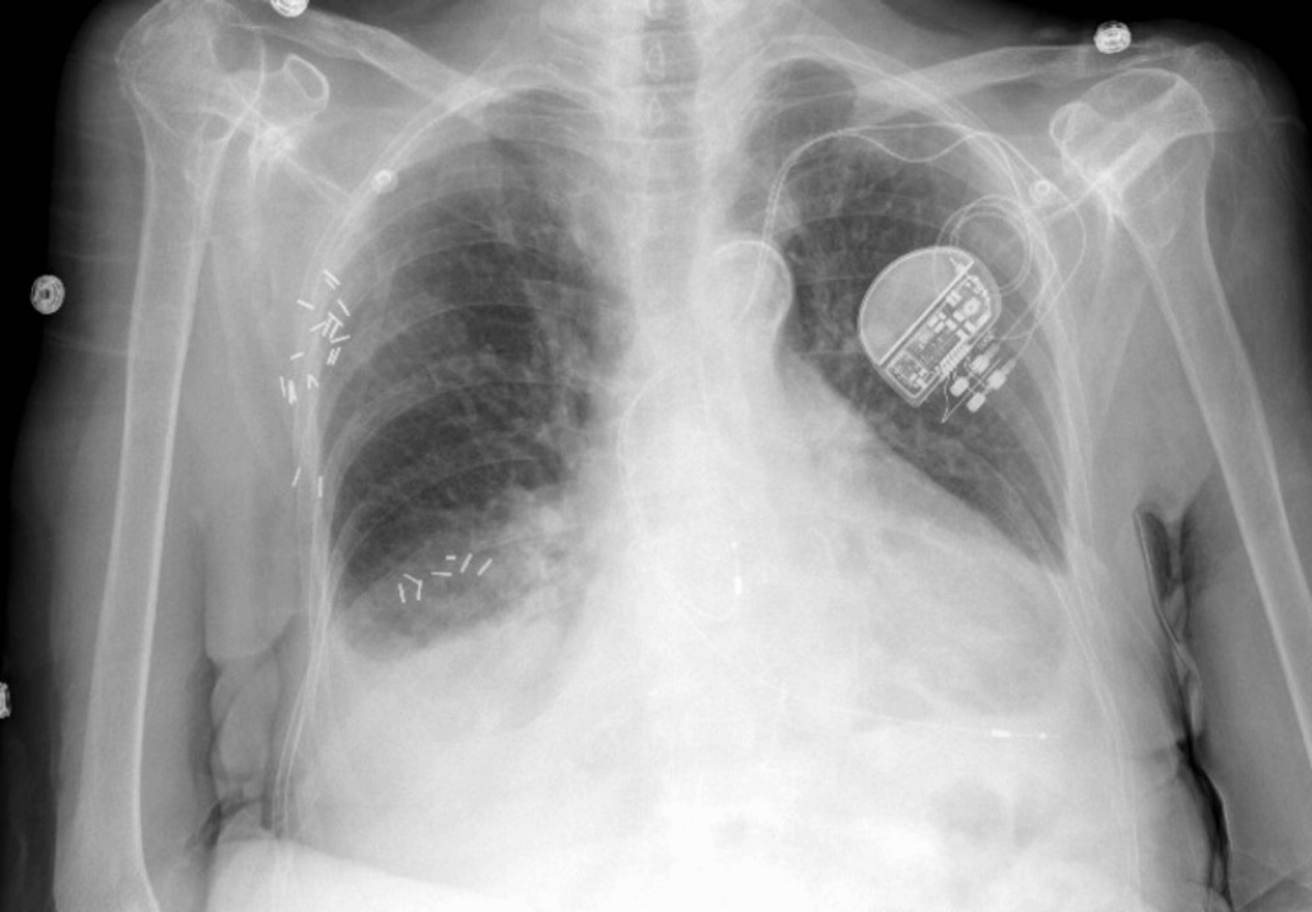
Chest X-ray showing mild pulmonary vascular congestion with opacity at bilateral lung bases.

A 98-year-old female with a past medical history of grade 3 heart failure with preserved ejection fraction was admitted with worsening dyspnea. Upon admission, SARS-CoV-2 RNA NAAT was detected as positive, and a chest radiograph revealed cardiomegaly, bilateral pleural effusions, and mild pulmonary vascular congestion (Figure 3). The patient was started on a five-day course of 200 mg IV remdesivir, 6 mg oral daily dexamethasone, and 40 mg furosemide. She required supplemental oxygen support via a high-flow nasal cannula and intermittent bilevel-positive airway pressure (BiPAP) throughout her admission.

The hospitalization was complicated by chronic pain in her extremities and difficulty weaning off oxygen support. Given her length of stay at the hospital, a registered dietitian evaluated her, determining 75%-100% completion of her meals without any other changes in appetite or dysphagia. There was a noted mild decline in cognitive function starting on hospital day 5, which improved by the time of discharge.

In the context of frequent hospital admissions and increasing frailty, with gradual, unspecified weight loss, goals of care discussions were initiated with the patient and her family. After careful consideration, the decision was made to transition the patient to hospice care at an external facility. The patient was discharged to a skilled nursing facility (SNF) following this 11-day hospitalization. Unfortunately, she passed away within two months of discharge.

Case 5

**Figure 4:**
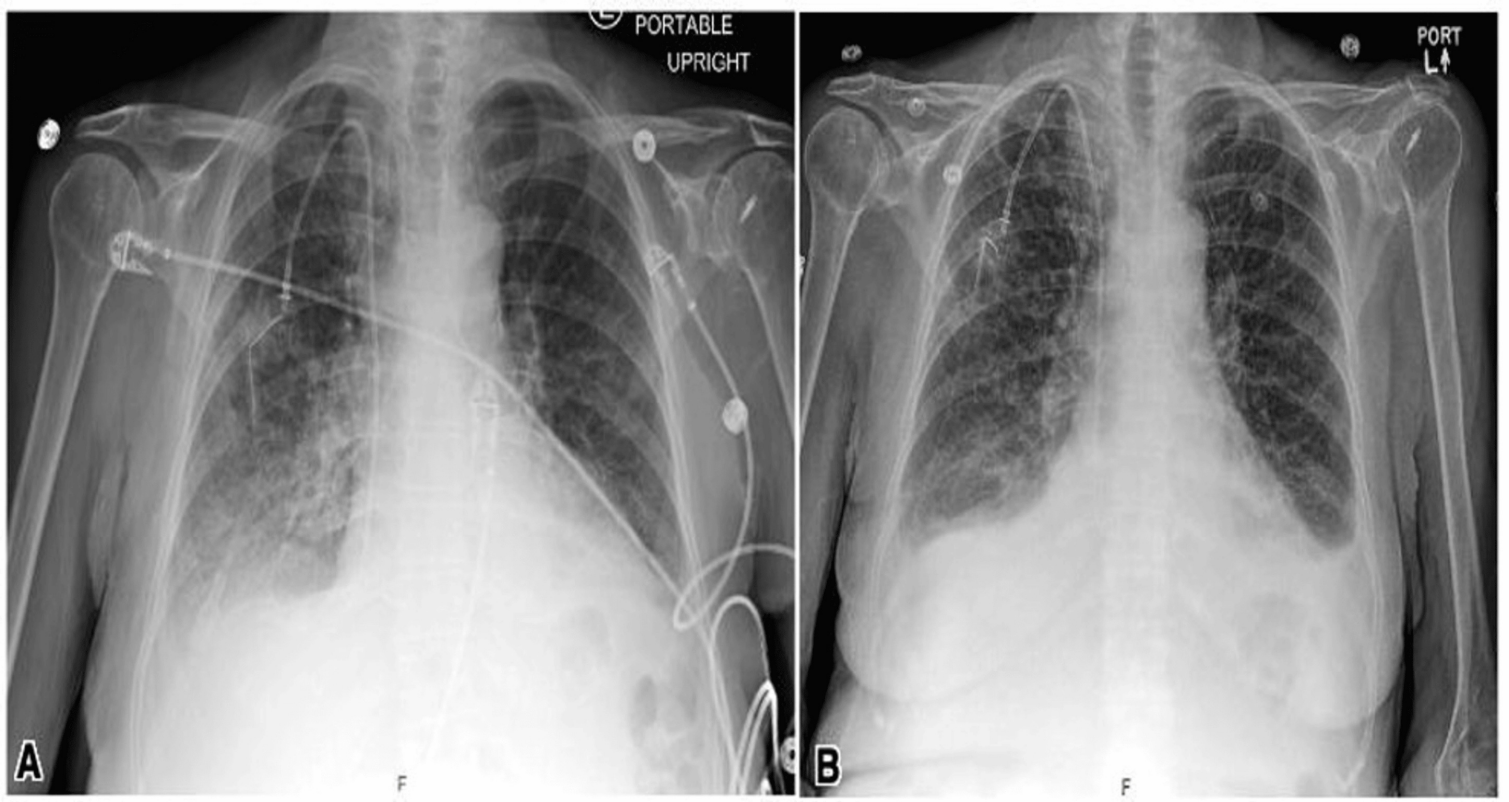
Chest X-ray on presentation showing pulmonary edema (A). Chest X-ray on hospital day 5 showed venous congestion and mild-to-moderate edema with bilateral small effusions (B).

An 80-year-old female with metastatic esophageal carcinoma on chemotherapy presented with fatigue, lightheadedness, and dark stool. Upon arrival, her hemoglobin was found to be severely low at 5.9 g/dL, without an obvious source of hemorrhage. Thrombocytopenia also worsened during her hospitalization, and although SARS-CoV-2 was initially negative, a repeat test returned positive on hospital day 4 after the onset of new dyspnea and cough. Radiographic evidence of pulmonary edema and effusions was noted, for which the patient was treated with remdesivir and dexamethasone (Figure 4).

During the hospital course, a rapid response was called for increased confusion from baseline, which was attributed to hospital-associated delirium. Additionally, a urinary tract infection was treated with ceftriaxone. The patient was kept NPO on day 1, pending evaluation by gastroenterologists, who determined that she did not require endoscopy and was safe to consume a clear liquid diet, which was eventually transitioned to a soft-bite-sized diet on hospital day 8. The registered dietitian determined that the patient was only consuming 50% of her meals throughout the course of her hospitalization, without any nausea, vomiting, or dysphagia. The patient was noted to have a decline in cognitive function from baseline starting on hospital day 1, which waxed and waned until discharge.

Upon discussions with the patient and family regarding the patient’s progressive cancer and poor prognosis, the decision was made to pursue comfort care and hospice, with a preference for palliative care services and a transition to home hospice if needed. After 14 days of hospitalization, the patient was discharged and, unfortunately, passed away within a month.

Case 6

**Figure 5:**
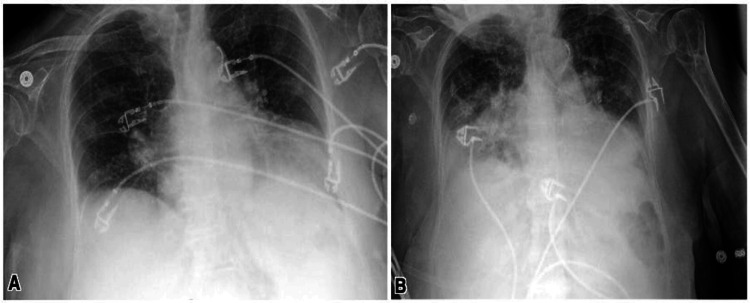
Chest X-ray on admission showed diminished lung volumes, clear lungs, and cardiomegaly. The aorta is noted to be tortuous and calcified (A). Chest X-ray on hospital day 3: new right lower lobe alveolar opacity consistent with pneumonia (B).

A 99-year-old female with a history of cerebrovascular accident (CVA), hypertension, and pituitary micro-adenoma presented with fever and hypotension. She was found to have a positive SARS-CoV-2 RNA NAAT test, and a chest radiograph showed clear lungs, decreased lung volume, and cardiomegaly (Figure 5). Due to increasing oxygen requirements, the patient was started on remdesivir and dexamethasone for five days. However, she developed worsening hypoxia, and a repeat chest radiograph showed a right lobe infiltrate, which was concerning for aspiration pneumonia. She was treated with broad-spectrum antibiotics for five days.

SLP evaluated the patient over multiple encounters starting on hospital day 1 and determined that the patient had severe oropharyngeal dysfunction, leading to an NPO diet. The patient had been tolerating a regular full diet without any concerns prior to this hospitalization. The cognitive decline from baseline was notable on hospital day 7 and worsened until the time of discharge. Due to the patient’s failure of the swallowing evaluation and oral feeding trials, and worsening COVID-19-associated encephalopathy, the family was consulted regarding percutaneous endoscopic gastrostomy (PEG) tube placement. However, it was decided that comfort feeds were a better option due to the poor evidence for long-term benefits in elderly patients. The code status was changed to do not resuscitate (DNR) and do not intubate (DNI).

The patient’s admission was further complicated by worsening liver function tests, likely due to refeeding or hepatic congestion. After 25 days of hospitalization, the patient was discharged to a SNF at the family’s request.

## Discussion

This case series presents six elderly patients, aged 80-99 years, diagnosed with COVID-19, each with multiple comorbidities and acute cognitive deterioration. All underwent multidisciplinary palliative care discussions, reflecting the complexity of their hospital courses. Notably, four patients (Patients 1, 2, 3, and 6) experienced worsening dysphagia, exacerbating frailty and leading to early palliative care decisions. Patient 1 had chronic oropharyngeal dysphagia following a prior stroke, worsened by COVID-19-associated encephalopathy. Patient 2, with Alzheimer’s disease, developed moderate-to-severe dysphagia, likely linked to encephalopathy as well. Patient 3 exhibited acute dysphagia and cognitive decline, also likely tied to COVID-19 encephalopathy. Patient 6, with a history of CVA, developed severe oropharyngeal dysphagia and cognitive deterioration, again pointing to COVID-19-related neurological effects. In contrast, Patients 4 and 5 experienced cognitive decline but no dysphagia.

Cognitive decline was a common feature in all cases, presenting at different times and with varying severity. These changes can be attributed, at least in part, to COVID-19-associated encephalopathy, a serious and underreported complication. Dysphagia affected swallowing function at various levels (oral, pharyngeal, and esophageal) in four patients, contributing to malnutrition, dehydration, and increased risk of aspiration pneumonia. These complications often prompted the initiation of palliative care, with most patients being discharged to hospice or a SNF with hospice planning. The rapid mental status deterioration seen in COVID-19-associated encephalopathy is likely due to factors such as hypoxia, systemic inflammation, and potential direct viral invasion of the central nervous system. This underscores the need for a coordinated, team-based approach involving physicians, nurses, therapists, and dietitians to manage both physical and emotional needs [10]. Dysphagia, in particular, represents a significant burden on quality of life, and its management should be integrated into palliative care strategies, including comfort feeding as part of compassionate care [11].

In-hospital delirium during COVID-19 hospitalization is associated with increased rates of functional disability and cognitive impairment over the months following discharge [12-15]. Delirium incidence varies widely among hospitalized COVID-19 patients, and factors such as ICU admission can influence these rates. Delirium is linked to poor short-term and long-term outcomes, including functional disability and cognitive impairment [16]. Predisposing factors for delirium in older adults include preexisting cognitive or functional disability, vision or hearing impairment, multimorbidity, and polypharmacy [17-20]. Evidence-based programs, like the Hospital Elder Life Program (HELP), have effectively reduced in-hospital delirium through reorientation, nonpharmacologic sleep protocols, early mobilization, use of hearing and visual aids, and treatment of dehydration [21,22]. The COVID-19 pandemic worsened the recognition and treatment of delirium due to staff shortages, the use of personal protective equipment, isolation policies, and increased use of sedative medications [23]. A renewed focus on implementing evidence-based interventions is needed to prevent delirium in vulnerable older adults hospitalized with COVID-19. Including family member participation in delirium prevention efforts, such as Family-Augmented-HELP, may enhance these efforts [24]. Healthcare personnel should recognize that older COVID-19 survivors who experience in-hospital delirium are at increased risk of long-term cognitive impairment and functional disability. Potential interventions for mental and functional impairment following COVID-19 should be considered.

Collaboration between palliative care and other disciplines remains suboptimal, even outside the context of COVID-19, and the pandemic has further emphasized the need to refine systems for initiating goals of care discussions [25]. Early involvement of palliative teams is essential for elderly patients with COVID-19-related decline, especially COVID-19 encephalopathy, as demonstrated in these cases. Palliative care provides valuable support for end-of-life planning and ensures a holistic approach to care, but healthcare systems still need greater integration of these services [26,27]. While the World Health Organization (WHO) provides clear guidelines for COVID-19 management, specific recommendations for palliative care are lacking [14]. This gap highlights the need to critically reassess current practices to improve care for patients with complex COVID-19-related conditions.

The main limitation of this study is possible selection bias and the lack of brain imaging to possibly support the diagnosis of COVID-19-associated encephalopathy, although no clear distinguishing findings have been reported to date. A larger-scale report is needed to support this possible association.

## Conclusions

This report highlights the clinical trajectory and deterioration of patients with COVID-19-associated encephalopathy. As new variants of COVID-19 continue to emerge, healthcare providers will face ongoing challenges in managing these complex cases. It is essential to look beyond the acute phase of COVID-19 to understand its long-term neurological impacts, enabling families to make informed care decisions. A multidisciplinary approach, including early involvement of palliative care, is crucial to ensure comprehensive patient management and to guide families through goal-setting and prognostic discussions. Collaboration across specialties will be key to providing optimal care in this evolving landscape.
